# LncRNA SNHG7 Mediates the Chemoresistance and Stemness of Breast Cancer by Sponging miR-34a

**DOI:** 10.3389/fonc.2020.592757

**Published:** 2020-11-24

**Authors:** Zhi-hua Li, Ni-si Yu, Qing Deng, Yulu Zhang, Yang-yang Hu, Gang Liu, Kedi Huang

**Affiliations:** ^1^ Department of Breast Surgery, Third Hospital of Nanchang, JiangXi Breast Specialist Hospital, Nanchang, China; ^2^ Key Laboratory of Breast Diseases in Jiangxi Province, Third Hospital of Nanchang, Nanchang, China; ^3^ Gynecology Department, Affiliated Hospital of Jiangxi University of Traditional Chinese Medicine, Nanchang, China; ^4^ Orthopedics Department, Third Hospital of Nanchang, Nanchang, China

**Keywords:** lncRNA SNHG7, chemoresistance, stemness, breast cancer, miR-34a

## Abstract

Chemoresistance is considered to be a major cause of the recurrence and metastasis of breast cancer (BC). LncRNA SNHG7 has been reported to be upregulated in breast cancer and to promote tumor progression and metastasis. Nevertheless, the function and potential regulatory mechanism of SNHG7 in BC drug resistance are still largely unclear. This study indicated that SNHG7 was highly expressed in chemoresistant BC tissues and cells. Upregulated SNHG7 might predict a low pCR rate and poor clinical outcome in BC patients. Knockdown of SNHG7 enhanced drug sensitivity and drug-induced apoptosis in chemoresistant BC cells. In terms of the mechanism, miR-34a was found to be a target of SNHG7 and its expression in breast cancer tissues and chemoresistant cell lines was negatively correlated with SNHG7 expression. Importantly, sh-SNHG7 upregulated miR-34a expression, reduced the percentages of CD44^+^/CD24^−^cells, and inhibited sphere-formation and stem cell factor (Oct4, Nanog, SOX2) expression. Functional loss experiments showed that the repressive effect of SNHG7 knockdown on BC cell stemness was partially reversed by transfection with miR-34a inhibitors. In summary, this study indicated that SNHG7 contributed to the chemoresistance of BC and mediated chemoresistance and cancer stemness by sponging miR-34a.

## Introduction

Breast cancer is one of the most prevalent cancers and the second leading causes of cancer-related death among women worldwide (1). Due to the continuous optimization of diagnostic methods and treatment measures, including surgery, chemotherapy and radiotherapy, the cure rate of BC has drastically improved during the past decade (2). Nevertheless, chemoresistance frequently occurs in advanced BC patients, which also leads to a poor prognosis for these patients (3). At present, doxorubicin and taxane are widely used in systemic chemotherapy of BC and their resistance usually implied the failure of the optimal treatment (4). Consequently, it is of great importance to sequentially elucidate the underlying mechanisms and to discover novel therapeutic targets to overcome chemoresistance in breast cancer patients.

LncRNAs are characterized as transcripts >200 nucleotides which have been widely focused on the regulation of gene expressions and biological process of many cancer phenotype in recent years (5). Numerous studies have indicated that lncRNAs are involved in the regulation of both intrinsic and acquired chemoresistance in breast cancer. LncRNA H19 induces paclitaxel resistance to ERα-positive breast cancer through epigenetic silencing of BIK gene (6) and leads to the propagation of doxorubicin resistance *via* delivery of exosomes to sensitive cells (7). LncRNA CASC2 mediates paclitaxel resistance to breast cancer through targeting miR-18a-5p/CDK19 axis (8). Knockdown of lncRNA-HOTAIR downregulates resistance of breast cancer cells to doxorubicin *via* the PI3K/AKT/mTOR signaling pathway (9). LINC00968 reduces drug resistance in breast cancer cells by blocking Wnt2/β-catenin signaling pathway through silencing WNT2 (10).

SNHG7 is a newly recognized lncRNA that is significantly upregulated in breast cancer (11). High expression of SNHG7 accelerates breast cancer tumorigenesis and progression by sponging miR-34a to activate EMT and Notch-1 pathway (12). Knockdown of SNHG7 was found to remarkably enhance cisplatin resistance in NSCLC cells by downregulating the PI3K/AKT pathway (13). Nevertheless, its exact role in the chemoresistance of breast cancer is still fully unclear. The present study aims to investigate the functions of SNHG7 in regulating chemoresistance in breast cancer and to preliminarily explore its potential molecular mechanism.

## Materials and Methods

### Patients and Tissue Specimens

From March 2018 to April 2019, 43 patients with advanced breast cancer who received at least six cycles of anthracycline- and taxane-based NAC at Third hospital of Nanchang were recruited for this study. All patients were female and pathologically diagnosed with primary invasive breast cancer by core needle biopsy prior to NAC. The median age of these patients was 49 (range, 27–64) years. All of the patients were treated according to the neoadjuvant chemotherapy standard in the 2018 NCCN breast cancer guidelines. Baseline clinical characteristics of all patients are shown in **Table 1**.

**Table 1:** Baseline clinical characteristics of all patients.

Variable	N	%
Menopausal status		
Pre-menopausal	27	62.8%
Post-menopausal	16	37.2%
Staging		
II-b	16	37.2%
III	27	62.8%
Histologic type		
Invasive ductal carcinoma	33	76.7%
Other types	10	23.3%
ER status		
Negative	22	51.2%
Positive*	21	48.8%
PR status		
Negative	27	62.8%
Positive*	16	37.2%
HER-2 status		
Negative	25	58.1%
Positive*	18	41.9%
Ki-67 index		
Negative	10	22.3%
Positive*	33	76.7%
Surgical types		
Breast conservation	2	4.7%
mastectomy	41	95.3%
Neoadjuvant Chemotherapy regimen		
EC or FEC	7	16.2%
CET or AC-T or PD +/-H	36	83.8%

*ER, PR Positive defined as “IHC positive cells ≥10%”; HER-2, Positive defined as “IHC 2+ OR Fish HER-2 gene amplified”; Ki-67, index positive defined as “IHC positive cells ≥30%”.

The patient response to NAC was assessed according to RECIST 1.1 criteria. Patients with complete response (CR) and partial response (PR) were classified as the response group. Patients with stabilization of disease (SD) and progressive disease (PD) were defined as Non-response group. In this study, no invasive tumor in both breast and lymph nodes were defined as pathological complete response (pCR) (14).

All tissue samples were fixed for 10 h in 10% neutral-buffered formalin before they were embedded in paraffin. A protocol for the use of tissue samples from patients and the procedures was approved by the Ethics Committee of Third Hospital of Nanchang. All participators signed an informed consent before enrollment.

### Cell Culture and Induction of Chemoresistance

Human normal breast epithelial cell MCF-10A and breast cancer cells (MCF-7 and MDA-MB-231 cells) were purchased from the Chinese Academy of Sciences (Shanghai, China). The cells were cultured in Dulbecco’s Modified Eagle’s Medium (DMEM, Gibco BRL) supplemented with 10% fetal bovine serum (FBS,Gibco) in a humidified atmosphere of 5% CO_2_ at 37°C.

To construct chemo-resistant breast cancer cells (MCF‐7/ADM and MDA‐MB‐231/PTX), their parent cells were each inducted by serial incremental concentrations of adriamycin and paclitaxel for at least 6 months. Then they were cultured in DMEM respectively containing with 4 μmol/L adriamycin and 10 µg/L paclitaxel to maintain the drug-resistant phenotype.

### RNA Extraction and qRT-PCR

Total RNA in the tumors before NAC and cells was extracted separately using RecoverAll™ Total Nucleic Acid Isolation kit for FFPE (Thermo Fisher Scientific, Inc., Waltham, MA, USA) and TRIzol Reagent (Invitrogen, Carlsbad, CA, USA) according to the manufacturer’s protocol. The RNA concentration in the FFPE samples and cells was measured and the RNA was used for reverse transcription and qRT-PCR. The detection of SNHG7 and miRNA-34a expressed in the clinical samples and cells was performed in a Step-One Plus Real-Time PCR System (Applied Biosystems, Carlsbad, CA). The qRT-PCR results were calculated using the 2^−ΔΔCt^ method and were respectively normalized to GAPDH and U6. The detail sequences of these primers used for qRT-PCR are listed in **Table 2**.

**Table 2:** Primer Sequences For RT-qPCR.

Gene	Sequence (5′-3′)
SNHG7	F:GTTGGGGTGTTGGCATTCTTGTT
	R:GCGCCCAATACGACCAAATC
miR-34a	F:AGCCGCTGGCAGTGTCTTA
	R:CAGAGCAGGGTCCGAGGTA
GAPDH	F:CGTCGCTAGCGATCGTTACA
	R:CTAAATGCTAGTCTTTACGA
U6	F:CTCGCTT CGGCAGCACA
	R:AACGCTTCACGAATTTGCGT

### Plasmid Transfection

Three small interfering RNAs against SNHG7 (si-SNHG7-1, si-SNHG7-2, and si-SNHG7-3), negative control (si-NC), miRNA-34a mimic (miR-34a), mimic control (NC-RNA), miRNA-34a inhibitor and miRNA-34a inhibitor control (inhibitor NC) were purchased from Ribobio (Guangzhou, China). A lentiviral vector expressing shRNA directed against SNHG7 (sh-SNHG7) and its scrambled shRNA (sh-NC), pcDNA3.1-SNHG7 vector (pc-SNHG7) and control empty pcDNA3.1 vector (pc-vector) were provided by GenePharma (Shanghai, China). These oligos and plasmids were transfected into BC cells using Lipofectamine 3000 Reagent (Invitrogen, Carlsbad, CA, USA). The transfection efficiency was assessed by qRT-PCR.

### Drug Resistance Assay

Transfected BC cells were inoculated into 96‐well plates and exposed to various concentrations of adriamycin and paclitaxel for 48 h. Subsequently, MTT assay was used to examine cell viability according to the manufacturer’s specification. To estimate adriamycin and paclitaxel sensitivity, half‐maximal inhibitory concentration (IC_50_) values were calculated based on the charted dose-response curve generated by GraphPad Prism 7.0 software.

### Apoptosis Assay

Cell apoptosis was assessed using FITC Annexin V Apoptosis Detection Kit (BD Biosciences, USA) in accordance with the manufacturer’s instructions. Transfected BC cells were cultured in 6-well plates and then treated with the indicated concentration of adriamycin or paclitaxel for 48 h. Subsequently, Cells were stained with Annexin V-FITC and PI for 30 min. The FACScan flow cytometry (BD Biosciences, San Jose, CA) was used to determine the ratio of apoptotic cells.

### Reporter Gene Assay

In all, 1×10^4^ HEK-293T cells were seeded into a 48-well plate and co-transfected with the SNHG7-luciferase reporter (10 ng) and miR-34a mimics (100 nmol/L) or NC-RNA using Lipofectamine 3000 (Invitrogen, USA). The luciferase activities were measured with a dual‐luciferase reporter gene assay system (Promega, Madison, WI, USA). Renilla luciferase acted as a reporter gene for normalized control.

### Tumorsphere Formation Assay

MCF-7/ADR cells with stable SNHG7 knockdown or empty vector (2× 10^3^/well) were grown in serum-free DMEM/F12 supplemented with 2% B27 (Gibco, Thermo Fisher), 20 ng/mL human epidermal growth factor (Peprotech, Rocky Hill, NJ, USA), 5 mg/mL insulin (Sigma-Aldrich, St. Louis, MO, USA), 1% penicillin and 0.4% bovine serum albumin (Sigma-Aldrich). After culturing for approximately 10 days, the tumorsphere formation were counted and quantified using a microscope (Olympus IX71; Olympus, Tokyo, Japan).

### CD44^+^/CD24^-^ Surface Marker Analysis by Flow Cytometry

MCF-7/ADR cells with stable SNHG7 knockdown or empty vector were suspended and seeded into 6-well plates with a density of 2x10^5^ cells/well. Then cells were washed with PBS with 2% FBS, incubated in PBS containing 2% FBS, anti-CD44-FITC (BD Biosciences) and anti-CD24-PE (BD Biosciences) for 30 min at 4°C. After staining, cells were washed three times with cold PBS buffer and analyzed by flow cytometry (BD Biosciences). The CD44^+^/CD24_-_ cells percentage was calculated using FACSDiVa software (BD Biosciences).

### Western Blot

The cells were lysed in RIPA lysis buffer supplemented with protease inhibitor mixture. The protein samples were separated by 12% SDS-PAGE gels and transferred to PVDF membranes (Thermo Fisher Scientific Inc., MA, USA). Then the membranes were immunoblotted with the primary antibody including anti-OCT4, anti-Nanog and anti-Sox2 (mouse polyclonal, 1:1000, Abcam, UK) overnight at 4°C. After washing in TBST, the membranes were incubated with the secondary antibodies conjugated by horseradish peroxidase (HRP) and the protein bands were visualized using an enhanced chemiluminescence imaging system (Clinx, Shanghai, China).

### Statistical Analyses

Statistical analysis was performed using SPSS version 18.0 software (IBM, Chicago, IL, USA) and GraphPad Prism (vision 7.0, USA). Differences between variants were compared using a Student’s t-test, Tukey’s test, and correlation analysis. Disease-free survival (DFS) between the groups was analyzed using the Kaplan-Meier method and log-rank test. The cut-off for the follow-up period was April 15, 2020. A value of P<0.05 was considered statistically significant.

## Results

### High Expression of SNHG7 Was Correlated With Chemoresistance

**Figure 1:**
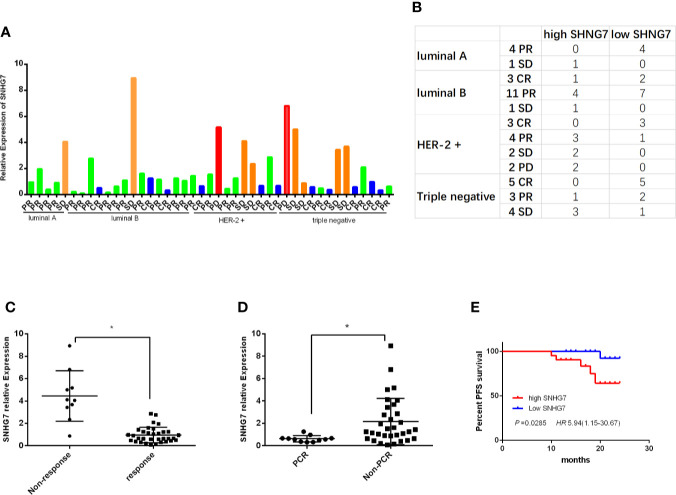
High expression of SNHG7 was correlated with an adverse response to NCT and poor RFS. **(A, B)** The relative SNHG7 expressions in BC patients with different molecular subtypes and different response to NCT, including complete response (CR), partial response (PR), stable disease (SD) and progression disease (PD). **(C)** The relative SNHG7 expression levels were associated with response to NCT. **(D)** The expression of SNHG7 in the pCR group compared with that in the Non-pCR group. **(E)** The Kaplan–Meier survival curve of patients with advanced breast cancer classified as low- and high-SNHG7 groups based on a median expression level of SNHG7. *P < 0.05.

The expressions of SNHG7 were detected in 43 cases of breast cancer tissue samples by RT-qPCR. The relative expression level of SNHG7 and the corresponding results of neoadjuvant chemotherapy in 43 patients were shown in **Figure 1A**. To evaluate the clinical significance of SNHG7 in advanced breast cancer, these patients were subdivided into low- and high-SNHG7 groups based on a median level of SNHG7 expression. The corresponding results between neoadjuvant efficacy and SNHG7 expression in BC patients with different molecular subtypes were shown in **Figure 1B**. Statistical analysis found that the expression of SNHG7 in response group (CR+PR) was lower compared with its in Non-response group (SD+PD) (P<0.001, **Figure 1C**). Moreover, the expression of SNHG7 in the pCR group was significantly lower than that in the Non-pCR group (p=0.019, **Figure 1D**).

Then the association between SNHG7 expressions and the clinicopathologic parameters of breast cancer patients were investigated. Statistical analysis showed that high SNHG7 levels strongly correlated with Tumor size (P=0.012), TNM stage (P = 0.016) and Ki-67 index (P = 0.037) (**Table 3**). Interestingly, Kaplan-Meier survival analysis revealed significantly DFS in high-SNHG7 groups was higher than its in low-SNHG7 groups (**Figure 1E**, P= 0.029). These data implied that upregulated SNHG7 might predict a low pCR rate and poor clinical outcome in advanced breast cancer patients.

**Table 3:** SNHG7 expressions and clinicopathologic parameters of BC patients.

Variable		SNHG7 expression		*P* value
		High(n=21)	Low(n=22)	
menopausal status				
Pre-menopausal		15	12	0.252
Post-menopausal		6	10
Tumor size				
≤5cm		3	11	0.012
>5cm		18	11
Staging				
II b		4	12	0.016
III		17	10
Histologic type				
Invasive ductal carcinoma		18	15	0.174
Other types		3	7
ER status				
Negative		12	10	0.443
Positive*		9	12
PR status				
Negative		14	13	0.607
Positive*		7	9
HER-2 status				
Negative		11	14	0.455
Positive*		10	8
Ki-67 index				
Negative		2	8	0.037
Positive*		19	14

* ER positive: >1%; PR positive: >1%; HER-2 positive: IHC 3+ OR Fish +; Ki-67 positive: ≧20%.

### SNHG7 Was Highly Expressed in Chemoresistant Breast Cancer Cells

**Figure 2:**
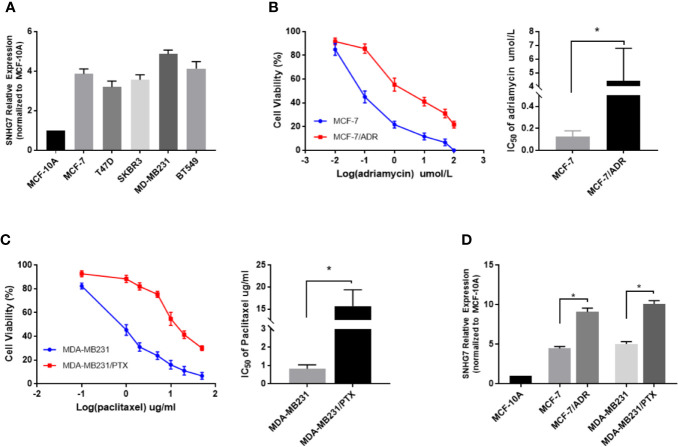
LncRNA SNHG7 was highly expressed in chemoresistant breast cancer cells. **(A)** The expression of SNHG7 in 5 breast cancer cell lines (MCF-7, T47D, SKBR3, MD-MB231, BT549) and normal human mammary gland epithelial cell line (MCF-10A) determined by qRT-PCR. **(B)** The viability of MCF-7/ADR and the parental MCF-7 cells, and the IC_50_ values of adriamycin were determined by MTT assay after exposure to different concentrations of adriamycin for 48 h. **(C)** The viability of MDA-MB-231/PTX cells and the parental MDA-MB-231 cells,and the IC_50_ values of paclitaxel were determined by MTT assay after exposure to different concentrations of paclitaxel for 48 h. **(D)** The lncRNA SNHG7 expression level was increased in chemoresistant cell lines (MCF-7/ADR and MDA-MB-231/PTX) compared with parental cell lines (MCF-7 and MDA-MB-231). *P < 0.05.

The expression of SNHG7 in 5 breast cancer cell lines (MCF-7, T47D, SKBR3, MD-MB231, BT549) and normal human breast epithelial cell line (MCF-10A) determined by qRT-PCR (**Figure 2A**). To investigate the expression level of SNHG7 in chemoresistant breast cancer cells, the resistance of these cells to adriamycin or paclitaxel was identified by MTT assay. The results showed that the IC_50_ values of adriamycin in MCF‐7/ADM cells and parental MCF‐7 cells were approximately 4.41 and 0.126, respectively (**Figure 2B**), while those of paclitaxel in MDA‐MB‐231/PTX cells and parental MDA‐MB‐231 cells were approximately 15.69 and 0.83, respectively (**Figure 2C**). The subsequent qRT‐PCR assay revealed the upregulation of SNHG7 expression in chemoresistant breast cancer cells compared with their respective parental cells (**Figure 2D**).

### Knockdown of SNHG7 Promoted the Sensitivity of Chemoresistant Breast Cancer Cells

**Figure 3:**
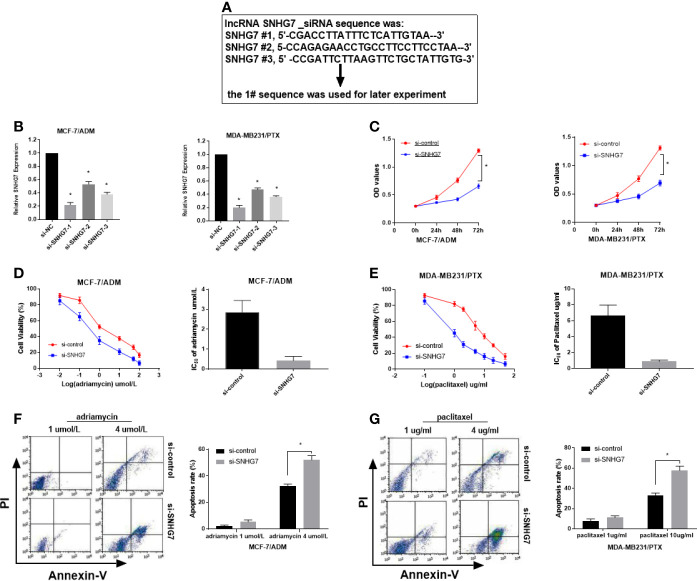
Knockdown of SNHG7 promoted the sensitivity of chemoresistant breast cancer cells. **(A)** Three interference sequences of lncRNA SNHG7. **(B)** qRT-PCR analysis was performed in MCF-7/ADR and MDA-MB-231/PTX cells transfected with SNHG7 siRNAs (si-SNHG7 #1, si-SNHG7 #2 or si-SNHG #3) or si-control. **(C)** Cell viability was evaluated in MCF-7/ADR and MDA-MB-231/PTX cells transfected with si-SNHG7 or si-control by MTT assay. **(D, E)** Cell viability was determined by MTT assay in transfected MCF-7/ADR and MDA-MB-231/PTX cells treated with various concentrations of adriamycin and paclitaxel. **(F, G)** Cell apoptosis was evaluated by flow cytometry analysis in transfected MCF-7/ADR and MDA-MB-231/PTX cells after treatment with adriamycin or paclitaxel, respectively. *P < 0.05.

To determine whether SNHG7 exerted any function in breast cancer, three synthesized small interference RNAs (si-SNHG7-1, si-SNHG7-2, and si-SNHG7-3) were transfected into MCF‐7/ADM and MDA‐MB‐231/PTX cells. QRT-PCR analysis indicated that the introduction of SNHG7 siRNAs weakened SNHG7 expression in MCF‐7/ADM and MDA‐MB‐231/PTX cells, especially in the si-SNHG7-1-treated group (**Figures 3A, B** **)**. Therefore, si-SNHG7-1 was defined as si-SNHG7 and was used in subsequent experiments. Dramatically, SNHG7-silencing decreased cell viability and enhanced adriamycin sensitivity in MCF-7/ADM cells and enhanced paclitaxel sensitivity in MDA-MB-231/PTX cells (**Figures 3C–E**). To further determine the role of SNHG7 in drug-induced apoptosis, flow cytometry analysis was performed in MCF-7/ADM cells after exposure to 4 µmol/L adriamycin and in MDA-MB-231/PTX cells after exposure to 10 µg/L paclitaxel. As expected, SNHG7 knockdown enhanced drug-induced apoptosis in MCF-7/ADM and MDA-MB-231//PTX cells (**Figures 3F, G** **)**. Collectively, SNHG7 knockdown facilitated drug sensitivity in breast cancer cells.

### SNHG7 Sponged MiR-34a in Chemoresistant Breast Cancer Cells

**Figure 4:**
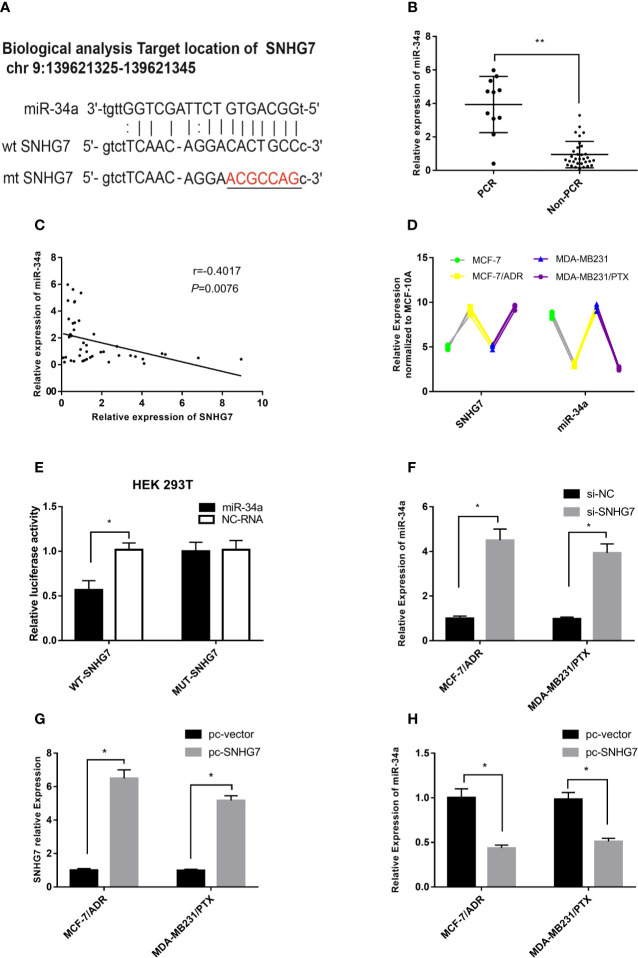
SNHG7 sponged miR-34a in chemoresistant breast cancer cells. **(A)** Bioinformatics analysis predicted that SNHG7 harbored miR-34a binding sites. **(B)** miR-34a expression was associated with pCR of breast cancer after NAC. **(C)** miR-34a was negatively correlated with SNHG7 expression in breast cancer tissues. **(D)** Compared with the parental cells, the expression of SNHG7 in MCF-7/ADR and MDA-MB-231/PTX cells was increased, while the expression of miR-34a was decreased. **(E)** A luciferase activity assay was performed after co-transfection of HEK-293T cells with a reporter plasmid and miR-34a. **(F)** miR-34a expression increased after transfection with si-SNHG7 into MCF-7/ADR and MDA-MB-231/PTX cells. **(G, H)** After MCF-7/ADR cells were transfected with the pcDNA3.1-SNHG7plasmid, the expression of SNHG7 increased, whereas it markedly reduced miR-34a expression. ^*^P < 0.05, ^**^P < 0.01 represent a statistically significant difference.

A bioinformatics analysis (http://starbase.sysu.edu.cn/index.php) revealed that putative complementary sequences of miR-34a in human SNHG7 were located on chromosome 9q34.3 and predicted miR-34a binding sites were found (**Figure 4A**). The expression of miR-34a was measured in clinical samples, and intriguingly, the results indicated that the expression of miR-34a in the pCR group was significantly higher than that in the Non-pCR group (p < 0.001, **Figure 4B**). Furthermore, a negative correlation was observed between SNHG7 and miR-34a expression in these breast cancer tissue samples (**Figure 4C**). Interestingly, compared with the parental cells, the expression of SNHG7 in MCF-7/ADR and MDA-MB-231/PTX cells was increased, while the expression of miR-34a was decreased (**Figure 4D**). A luciferase reporter assay was performed to evaluate the direct interaction between SNHG7 and miR-34a. The results showed that transfection of cells with miR-34a mimics significantly decreased WT-SNHG7-luciferase activity but that transfection with MUT-SNHG7 did not (**Figure 4E**). In MCF-7/ADM cells, SNHG7 silencing increased miR-34a expression (**Figure 4F**), while SNHG7 transfection markedly reduced miR-34a expression (**Figures 4G, H** **)**. These results suggested that SNHG7 directly targeted miR-34a and negatively regulated miR-34a expression.

### SNHG7 Modulated Chemoresistance and Cancer Cell Stemness Partially *via* MiR-34a

**Figure 5:**
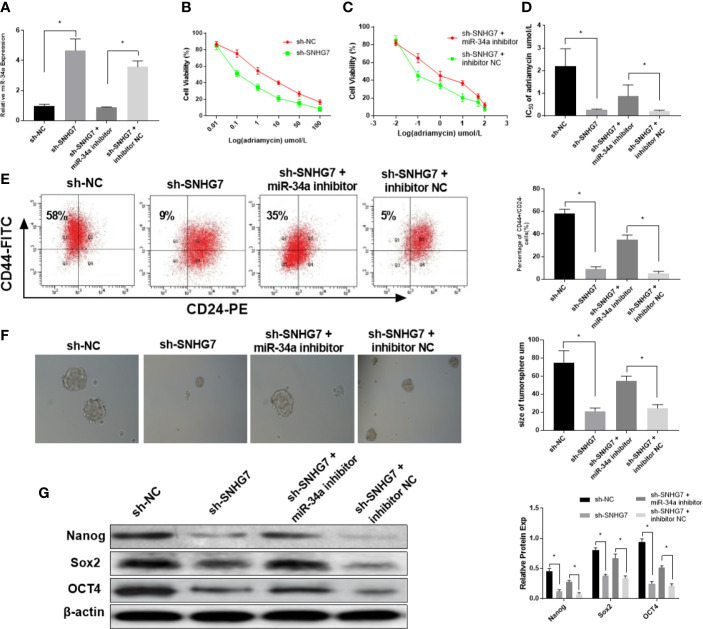
SNHG7 modulated chemoresistance and cancer cell stemness partially *via* miR-34a. **(A)** The expression of miR-34a in MCF-7/ADR cells was detected in MCF-7/ADR cells transfected with sh-NC, sh-SNHG7, sh-SNHG7+miR-34a inhibitor and sh-SNHG7+ inhibitor NC by qRT-PCR. **(B–D)** The IC50 of adriamycin in MCF-7/ADR cells transfected with sh-NC, sh-SNHG7, sh-SNHG7+miR-34a inhibitor and sh-SNHG7+ inhibitor NC was determined by MTT assay. **(E)** The percentages of CD44+/CD24- MCF-7/ADR cells transfected with sh-NC, sh-SNHG7, sh-SNHG7+miR-34a inhibitor and sh-SNHG7+ inhibitor NC was determined by flow cytometry analysis. **(F)** The diameters of sphere-forming cells in the sh-NC, sh-SNHG7, sh-SNHG7+miR-34a inhibitor and sh-SNHG7+ inhibitor NC groups were determined by sphere formation assay. **(G)** The protein expression levels of Nanog, SOX2, and OCT4 in MCF-7/ADR cells transfected with sh-NC, sh-SNHG7, sh-SNHG7+miR-34a inhibitor and sh-SNHG7+ inhibitor NC were measured by western blot. *P < 0.05 represent a statistically significant difference.

To further investigate the mechanism of SNHG7 in chemoresistance, MCF-7/ADR cells were co-transfected with SNHG7 shRNA and miR-34a inhibitors. The results indicated that transfection of with SNHG7 shRNA upregulated miR-34a expression in breast cancer cells, which was exceptionally reversed by miR-34a inhibitors (**Figure 5A**). An MTT assay revealed that knockdown of SNHG7 facilitated drug sensitivity of breast cancer cells, but nevertheless, the inductive effect of SNHG7 inhibition on drug sensitivity of breast cancer cells was patently abolished by miR-34a downregulation (**Figures 5B–D**). The presence of breast cancer stem cells is one of the most important reasons for chemoresistance and recurrence. Flow cytometry analysis showed that the percentages of CD44^+^/CD24^–^ cells were decreased in SNHG7-deficient MCF‐7/ADR cells, while this downward trend was partially reversed after treatment with miR-34a inhibitors (**Figure 5E**). A sphere formation assay indicated that the diameters of sphere‐forming cells in the sh-SNHG7 group were smaller than those of sphere-forming cells in the NC-vector group, but the spheres were relatively restored to their original size after transfection with miR-34a inhibitors (**Figure 5F**). Furthermore, the protein expression levels of cell stemness markers, including Nanog, SOX2, and OCT4, were reduced in sh‐SNHG7 MCF‐7/ADR CSCs, but also recovered as a result of miR-34a silencing (**Figure 5G**). These results indicated that lncRNA SNHG7 mediated drug resistance and cancer stemness by sponging miR‐34a.

## Discussion

LncRNA SNHG7 is located on chromosome 9q34.3, has a length of 2157 bp, and has been demonstrated to act as an oncogene in tumors. Moreover, its dysregulation has been found to be associated with carcinogenesis and progression of several cancers, such as lung cancer (15), gastric cancer (16), glioblastoma (17) and colorectal cancer (18). The expression of SNHG7 was upregulated in breast cancer and was positively correlated with tumor stage, lymph node metastasis and distant metastasis (19). SNHG7 contributes to breast cancer tumorigenesis and progression by sponging to regulate the miR-34a/Notch-1 axis (12), miR-381 (11), and miR-186 (19). However, whether SNHG7 is involved in chemoresistance in BC remains unclear.

Taxane- and anthracycline-based regimens are effective treatment options for advanced BC and are widely used in clinical practice. Chemoresistance to these drugs is believed to be the main obstacle for the treatment of breast cancer (20). In our study, we further found that the expression of SNHG7 was upregulated in chemoresistant breast cancer, which was also associated with an adverse response to NAC and poor RFS. Knockdown of SNHG7 decreased cell viability, enhanced drug-induced apoptosis and facilitated drug sensitivity in breast cancer cells. Similarly, Chen et al. (13) reported that knockdown of SNHG7 remarkably enhanced cisplatin resistance in NSCLC cells, which manifests as decreased cell viability, migratory and invasive rates, DNA synthesis capacity, and promotion of apoptosis.

Emerging evidence states that lncRNA SNHG7 sponges miR-34a-5p to promote tumor progression, EMT and invasion in different cancers (12, 21–23). In our study, using a luciferase reporter assay and correlation analysis in NAC clinical samples, we confirmed again that miR-34a-5p is a target miRNA of SNHG7. In addition, miR-34a-5p was also demonstrated to be directly combined with SNHG7 *via* a lncRNA gain/loss-of-function strategy. MiR-34a expression in human breast cancer is associated with drug resistance through targeting Bcl-2, CCND1 and Notch 1 (24, 25). MiR-34a modulated breast cancer stemness and drug resistance through GSK3/β-catenin signaling (26). Our results further verified that knockdown of SNHG7 facilitated drug sensitivity of breast cancer cells through miR-34a overexpression.

The presence of breast cancer stem cells (BCSCs) is one of the most important reasons for chemoresistance and recurrence. A previous study supported the finding that miR-34a acted as a tumor suppressor and can separately reduce the stemness of BCSCs (27, 28). Moreover, miR-34a can target PRKD1 to overcoming cancer stemness and drug resistance in human breast (26) and hTERT promoter-driven VISA delivery of miR-34a (TV-miR-34a) can significantly inhibit the tumor-initiating properties of long-term-cultured BCSC *in vitro* and reduced the proliferation of BCSC *in vivo *(29). Accumulated data have indicated that stable changes in the expression of SOX2, OCT4 and Nanog affect the self-renewal capacity of CSCs (27). Therefore, the role of SNHG7 in the stemness of breast cancer cells was investigated in this study. The results indicated that knockdown of SNHG7 decreased the percentages of CD44^+^/CD24^−^cells, inhibited sphere-formation and stemness factors (Oct4, Nanog, SOX2) expression. Further functional loss experiments showed that the repressed effect of SNHG7 knockdown on BC stemness was achieved by miR-34a.

In summary, the results of the present study indicated that high expression of SNHG7 may be a predictor of chemoresistance in breast cancer. Furthermore, the knockdown of lncRNA SNHG7 reduces drug resistance and inhibits stemness in breast cancer cells *via* miR‐34a, which indicates that lncRNA SNHG7 may be a potential therapeutic target to overcome chemoresistance in breast cancer patients.

## Data Availability Statement

The raw data supporting the conclusions of this article will be made available by the authors, without undue reservation.

## Ethics Statement

The studies involving human participants were reviewed and approved by the Ethics Committee of Third Hospital of Nanchang. The patients/participants provided their written informed consent to participate in this study.

## Author Contributions

All authors contributed to the article and approved the submitted version.

## Funding 

This work was supported by the National Natural Science Foundation of China (Contract grant numbers: 81860546).

## Conflict of Interest

The authors declare that the research was conducted in the absence of any commercial or financial relationships that could be construed as a potential conflict of interest.

## Abbreviations

SNHG7, Small Nucleolar RNA Host Gene 7; BC, Breast cancer; NAC, Neoadjuvant chemotherapy; RFS, Recurrence-free Survival; NSCLC, Non-small cell lung cancer; RECIST, Response Evaluation Criteria in Solid Tumors; DMEM, Dulbecco’s Modified Eagle’s Medium; STR, Short tandem repeat; pCR, Pathological complete response; FFPE, Formalin-fixed paraffin embedded; OS, Overall Survival; DFS, Disease-Free Survival; HR, Hazard ratio; CSCs, Cancer stem cells.
